# Supplementary figures and images for: CNS-Tau Specific Antibodies Illuminate Disease Signatures Across Tauopathies
Source: bioRxiv. 2025 Sep 17:2025.09.14.676119. Preprint. [Version 1] doi: 10.1101/2025.09.14.676119 (PMC12458136; doi:10.1101/2025.09.14.676119)

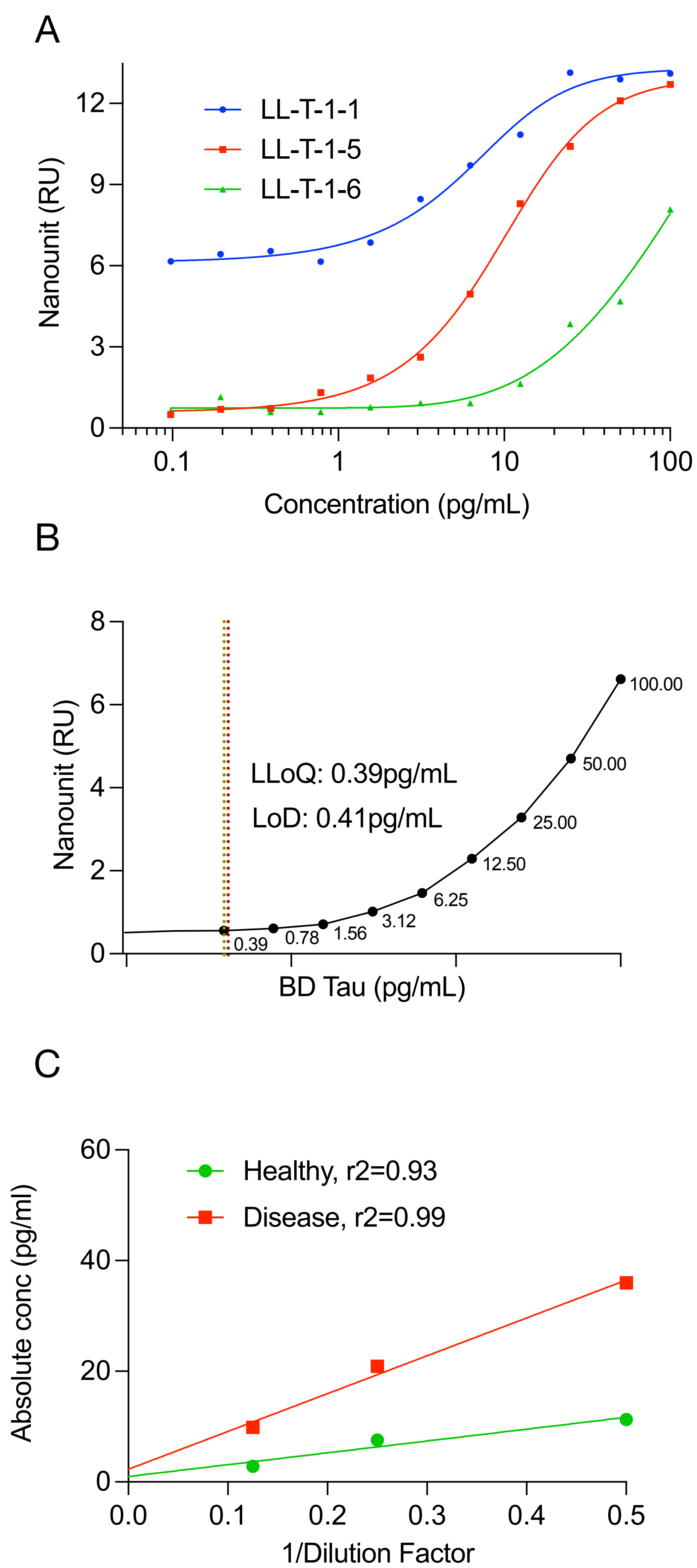

Fig.S1

Supplement: Supplement 1 — Supplementary Figure 1. Development of the plasma CNS-tau assay using the NanoMosaic platform. (A) Comparison of three LL-T-1 clones used as capture antibodies (paired with Tau-12 as detector) for recombinant Tau-441, evaluating signal-to-noise ratios. (B) Standard curve generated using LL-T-1–5 as the capture and biotinylated Tau-12 as the detector antibody, calibrated with recombinant Tau-441. (C) Dilution linearity of the plasma CNS-tau assay across two human plasma samples at 2×, 4×, and 8× dilutions, demonstrating strong linear response. [file media-1.pdf]
